# Impact of a peer-led, community-based parenting programme delivered at a national scale: an uncontrolled cohort design with benchmarking

**DOI:** 10.1186/s12889-022-13691-y

**Published:** 2022-07-18

**Authors:** Crispin Day, Joshua Harwood, Nadine Kendall, Jo Nicoll

**Affiliations:** 1 Centre for Parent and Child Support, South London and Maudsley NHS Foundation Trust, Michael Rutter Centre, De Crespigny Park, Camberwell, London, SE5 8AZ UK; 2 Harwood Child Psychology, London, UK; 3 Department of Psychology, Institute of Psychiatry Psychology & Neuroscience, King’s College London, London, UK

**Keywords:** Parenting, Child development, Behavioural disorders, Implementation science, Dissemination

## Abstract

**Background:**

Childhood behavioural problems are the most common mental health disorder worldwide and represent a major public health concern, particularly in socially disadvantaged communities. Treatment barriers mean that up to 70% of children do not receive recommended parenting interventions. Innovative approaches, including evidence-based peer-led models, such as Empowering Parents Empowering Communities’ (EPEC) Being a Parent (BAP) programme, have the potential to reduce childhood difficulties and improve parenting if replicable and successfully delivered at scale.

**Method:**

This real-world quasi-experimental study, with embedded RCT benchmarking, examined the population reach, attendance, acceptability and outcomes of 128 BAP groups (*n* = 930 parents) delivered by 15 newly established sites participating in a UK EPEC scaling programme.

**Results:**

Scaling programme (SP) sites successfully reached parents living in areas of greater social deprivation (*n* = 476, 75.3%), experiencing significant disadvantage (45.0% left school by 16; 39.9% lived in rental accommodation; 36.9% lone parents). The only benchmarked demographic difference was ethnicity, reflecting the greater proportion of White British parents living in scaling site areas (SP 67.9%; RCT 22.4%). Benchmark comparisons showed scaling sites’ parent group leaders achieved similar levels of satisfaction. Scaling site parent participants reported substantial levels of improvement in child concerns (ES 0.6), parenting (ES 0.9), parenting goals (ES 1.2) and parent wellbeing (ES 0.6) that were of similar magnitude to RCT benchmarked results. Though large, parents reported lower levels of parenting knowledge and confidence acquisition compared with the RCT benchmark.

**Conclusion:**

Despite common methodological limitations associated with real-world scaling evaluations, findings suggest that this peer-led, community-based, parenting approach may be capable of successful replication at scale and may have considerable potential to improve child and parenting difficulties, particularly for socially disadvantaged populations.

**Supplementary Information:**

The online version contains supplementary material available at 10.1186/s12889-022-13691-y.

## Background

Childhood behavioural disorders, characterised by persistent aggressive, oppositional and defiant behaviours, are the most common mental health disorder worldwide, representing a growing public health concern with poor outcomes persisting into adulthood [1–5]. In the UK, 4.6% of children aged 5–19 years meet behaviour disorder criteria [6], and a further 15–20% have significant, subclinical difficulties. UK lifetime estimated costs range from £85,000 per moderate case to £260,000 per severe case [7]. Behaviour disorders are twice as common in disadvantaged neighbourhoods and communities, and between two to four times more likely in families living in poverty, receipt of disability and other welfare benefits [7].

Up to 70% of children with behavioural disorders do not receive recommended interventions [8]. Barriers to care include insufficient service capacity, limited availability of evidence-based intervention, complex access arrangements, long waiting times, family stigma, and poor lay mental health knowledge. Typically, interventions are offered by highly trained specialist mental health professionals with postgraduate level education, delivered at clinic and healthcare facilities. Evidence-informed approaches are more common compared to the use of manualised, evidence-based methods frequently used in research trials. Manualised methods usually specify intervention contents, format and methods in predetermined written protocols. These service and practice barriers hinder problem identification, parent help-seeking, and limit the capability of routine services to deliver effective care at sufficient scale to substantially reduce prevalence and impact, particularly for low income, Black and Minoritised families [9, 10].

Group-format evidence-based parenting approaches are effective when tested in highly controlled experimental trial conditions and are recommended as the first line response [11–13]. These approaches can maintain performance in real world conditions but financial cost and almost exclusive dependence on delivery by highly trained and specialist practitioners inhibit availability at the scale required to meet the mental health needs of children and young people [14–17].

There is significant concern about the continuing failure to meet child and family need. The use of more innovative approaches, including peer-led models, has been recommended [18]. Less is known about the delivery at scale of peer-led approaches [19, 20], in which manualised parenting approaches are delivered by trained and quality assured non-professionals with the aim of increasing access, acceptability and reach, particularly for low income and Minoritised families. If effective at scale, the lower associated service costs of these approaches can potentially increase capacity and reduce the treatment gap.

Benchmarking is potentially an efficient, low-cost method that can be used to systematically examine the performance of evidence-based approaches as they travel from definitive and pragmatic trials to novel settings and real-world conditions [21–23]. Benchmarking can not only compare outcomes but can also assess target population reach and acceptability. As a relatively novel approach, benchmarking has been used to assess interventions in acute medicine and adult mental health but rarely in the field of child mental health and parenting.

### Scaling-up and scaling-out evidence-based approaches

Real world replication is complicated, unpredictable and success is not guaranteed [24, 25]. Scaling-up involves dissemination based on established conditions in which new providers typically adhere to pre-determined methods and protocols that are intended to reproduce trial outcomes. Pre-determined trial conditions may be challenging to reproduce in real world settings. Scaling-out, on the other hand, refers to replication in conditions that differ from original trial conditions [24], potentially offering great flexibility but risking variations in population reach, delivery, and fidelity that can undermine performance [25, 26].

### Empowering Parents Empowering Communities scaling programme

Empowering Parents Empowering Communities (EPEC) is a task sharing, peer-led parenting approach. Its group-based parenting course format is consistent with policy recommendations and intended to build social support between participants, optimise impact, and lower unit cost. EPEC is delivered in local, community locations and the programme uses high visibility, pro-active local outreach campaigns to engage parents. Within these targeted community locations, an open access approach is typically used, rather than formal referral. The peer-led format is associated with high levels of parent engagement, acceptability and reduced stigma. Randomised control trial and field evidence shows that EPEC *Being a Parent* successfully reaches socially disadvantaged and Minoritised parents of children aged 2–11 years, is highly acceptable, and produces significant improvements in child behaviour, positive parenting and parental concerns when delivered by peer parent group leaders (PGLs) recruited from within target populations, directly trained and supervised by EPEC developers [19, 20].

Funded by the UK Early Years Social Action Fund, NESTA and Department for Culture, Media and Sport, the EPEC Scaling Programme examined the scalability of the *Being a Parent* parenting course in 15 newly established EPEC Hubs located in socially disadvantaged areas across England. The funders specified a narrower target population of parents of children aged 2–5-years. The Programme scaled-up established EPEC methods, including its peer-led approach, manualised training, quality assurance procedures, and scaled-out by testing delivery in new service organisations types, such as local authorities and voluntary organisations, rural as well as new urban settings, inclusion of socially disadvantaged populations that potentially differed in characteristics from previous research and field trials, and novel hub setup, parent group leader recruitment, and implementation support methods.

The evaluation reported in this paper had two aims:

1. To examine the parent population reach, parent attendance and acceptability across Scaling Programme sites and compare these with established *Being a Parent* RCT benchmarks.
2. To evaluate the impact of the *Being a Parent* parenting course across Scaling Programme sites on child, parent and parenting outcomes and compare these with established RCT benchmarks.

## Method

### Design

A pragmatic cohort design incorporating a benchmarking comparison derived from previously published RCT results was used [19, 20, 27]. Demographic information and outcome measures were collected at the beginning (Time 1) and end (Time 2) of the *Being a Parent* parenting course, acceptability data were collected at Time 2. Attendance data were collected throughout each parenting course. Over the course of the evaluation period, 1135 parents attended a *Being Parent* information session, 930 (89.9%) participated in the parenting course and 684 parents completed it. Of the parents participating in the course, 730 (78.4%) completed Time 1 measures and 405 (55.5%) completed Time 2 measures.

### Participants

#### New EPEC Hubs

Fifteen EPEC hub host organisations: 10 local authorities, three NHS Trusts and two charitable organisations were selected because of compatibility between their local strategic priorities, operational resources, parenting and peer expertise, and population needs, and EPEC aims and programme theory, see Appendix 1: Figure A1. Over the 18-month duration of the Scaling Programme, hubs delivered 128 *Being a Parent* parenting groups from 97 different venues. Sixty-five venues (67.0%) were in the lowest third of the most deprived UK neighbourhoods, with 29 venues (29.9%) in the 10% most deprived areas [28]. 

#### Participant parents

Parents were eligible for the *Being a Parent* course when they were a primary parental caregiver who: 1) reported difficulties in managing behaviour of an index child aged 2–5 years, and 2) expressed concerns about their parenting. Families were excluded when the parent: 1) had insufficient English to complete evaluation measures, 2) could not attend weekly course sessions and therefore unlikely to fully participate in the *Being a Parent* course, 3) was not living with the index child and unlikely to have sufficient contact to implement parenting skills acquired during the course, and 4) the child experienced significant neurodevelopmental difficulties, such as autism, for which parents were likely to require specialist parenting intervention.

### Measures

#### Demographic information

Included parent age, ethnicity, first language, parent status, educational qualifications, housing and employment status.

#### Clinical outcomes

In families with more than one child aged 2–5 years, participants completed measures on the child about whom they had most significant concerns.

##### **Concerns About My Child** (CAMC, [19])

An idiographic measure of parental perception of child difficulties, previously used in *Being a Parent* trial evaluation. Parents rate up to three main child emotional and behavioural concerns from 0 (not concerned at all) to 100 (could not be more concerned). Concerns were categorised into five domains: Conduct Problems, Parent–Child Relationship and Communication Difficulties, Self-Regulation, Emotional Distress and Other.

##### **Arnold O’Leary Parenting Scale** (PS, [29])

Previously used in the *Being a Parent* trial, this 30-item questionnaire assesses dysfunctional parental discipline styles for children aged 2–16 years, yields a total score and parental verbosity, over-reactivity and laxness subscales. Lower scores indicate more positive parenting skills. Total score ≥ 3.2 differentiates between clinic and non-referred children. In this study, there was good internal consistency for the total score (α = 0.77).

##### **My Parenting Goals** (MPG)

An idiographic measure of up to two personal parenting goals, using a visual analogue scale from 0 (could not be further from achieving my goal) to 100 (goal completely achieved).

##### **Short Warwick Edinburgh Mental Wellbeing Scale** (SWEMWBS, [30])

A seven-item parent wellbeing measure each rated on a 5-point Likert scale. High scores represent greater mental wellbeing. SWEMWBS is sensitive to change and the full version has been used in evaluations of parenting programmes. It had good internal consistency, α = 0.85. Raw SWEMWBS scores were transformed to allow comparisons with national survey data.

#### Being a Parent acceptability and satisfaction

##### **Treatment Acceptability Rating Scale** (TARS—19)

This 12-item self-report questionnaire, previously used in *Being a Parent* trial, uses a 4-point Likert scale to assess, (i) parenting knowledge, skills and confidence acquired (TARS KSC—4 items yield total score 4–16) and (ii) course satisfaction and quality (TARS SQ—5 items yield total score 5–20). Higher scores indicate greater acceptability and satisfaction. Three free-text items cover helpful and unhelpful participant experiences.

#### Being a Parent attendance

Parent attendance, non-attendance, cancellation and drop-out was recorded prospectively by parent group leaders for each *Being a Parent* course using a secure online spreadsheet that generated an anonymised identifier for each parent.

### **EPEC *Being a Parent* Scaling Programme**

This comprised three inter-related phases:

*Phase 1: Hub engagement and initial set up (0–6 months)*: Hub site selection, licence agreement, staff appointment, initial 3-day hub familiarisation training covering *Being a Parent* quality standards and functions, staff roles and responsibilities, and evaluation.

*Phase 2: Hubs organisation (3-9 months)*: Hub staff training in *Being a Parent* manualised content and methods (4-days), PGL recruitment and training, supervision and quality assurance (3-days), and engagement of local stakeholders and communities. Each hub used existing local family, service and community networks to recruit an initial cohort of 12–16 PGLs, who completed a certified 60-hr training covering: (1) *Being a Parent* knowledge, methods and skills, (2) child development, parenting and family resilience, (3) group dynamics and facilitation skills, and (4) local safeguarding procedures. Participants completed an assessed portfolio and supervised practice prior to certification.

*Phase 3: Hub implementation (6–18 months)*: Each hub established pathways to engage local parents, ran ‘coffee morning’ information sessions, organised a rolling programme of supervised *Being a Parent* groups. National EPEC consultants used manualised quality standards to appraise hub implementation, problem-solve and support site scaling using ongoing digital and face-to-face contact and quarterly collaborative Hub learning and exchange events equivalent to one-day per month.

Findings are available elsewhere that describe the acceptability and impact of the training provided to hubs during the Scaling Programme and the demographic characteristics and training outcomes for parent group leaders recruited by hubs [31]. Working in pairs, 159 certified parent group leaders delivered *128 Being a Parent* courses, each co-delivering one to four groups.

#### Being a Parent Course

The *Being a Parent* course consisted of eight, two-hour sessions, with on-site crèche facilities, for 8–12 parents. It used large and small group discussion, information sharing, demonstrations, practice and homework to enable parents to acquire key parenting knowledge, understanding and skills based on child development, social learning, attachment, systems, family relations, communication and reflective function concepts. This content covered parent wellbeing and expectations; understanding children’s needs, emotions and behaviour; child-led play, listening and communication; praise and encouragement; and positive discipline strategies. Course completion was based on attendance of five or more sessions [19].

Participants were recruited through direct parent contact, word of mouth, recommendation by existing community and specialist services, and printed information and posters available in key family community locations, such as children’s centres and local schools. Prior to enrolment, prospective parents were invited to an introductory ‘coffee morning’ information session. Course fidelity and quality assurance, designed to monitor and maintain course norms, consolidate PGL skills, provide support and monitor safety, was undertaken through 1) PGL fortnightly supervision and (2) supervisor fortnightly observation of course delivery and practice.

### Procedure

After registration and prior to the first course session, participant parents received a link to a secure online Qualtrics survey portal to confirm consent and complete Time 1 measures using a uniquely generated anonymised identifier. Time 2 data was collected via a second Qualtrics link sent prior to the final course session. Online data was returned digitally directly to the Scaling Programme evaluation team at King’s College London. Parents could withdraw from the evaluation without it affecting their participation in the parenting course. The study team did not have the resources to follow-up parents who did not complete Time 2 measures.

### Service evaluation and informed consent

The aims of this evaluation met criteria for service evaluation rather than research or audit [32, 33]. It was designed and conducted with the sole purpose of defining or judging the service provided by the national EPEC dissemination team. The service evaluation did not explore nor seek to undertake an experiment to investigate or establish broader evidence about wider research issues related to parenting interventions nor implementation science.

Each parent participating in the service evaluation provided consent prior to completing the evaluation measures. Data was anonymised using individual parent codes. The service received by the parents was not conditional nor affected by taking part in the evaluation.

### Analysis plan

A cohort analysis using a merged dataset from across participating sites was conducted. An intention to treat analysis was not planned because of the increased likelihood of substantial data loss in large scale community evaluations of this type [15, 34]. No between site comparisons were planned due to the limited sample sizes available for individual sites.

Statistical analysis was mainly descriptive using means and SD for continuous demographic, acceptability and attendance data, and medians and range for skewed data. Frequencies and proportions were used to describe categorical variables. Continuous variables were compared using independent sample t-tests and proportion variables were compared using chi squared analysis. Clinical outcome change scores (Time 2 minus Time 1) were calculated for all measures and t-tests. Cohen’s d effect sizes ($a$=0.05) were calculated as follows:

$$\mathrm{Cohen}'\mathrm s\;{\mathrm d}_{\mathrm{av}}=\frac{\overline{x\mathit1}-\overline{x\mathit2}}{\sqrt{\displaystyle\frac{SD_11^2+SD_2^2}2}}$$

To reduce potential bias, univariate outliers were removed pairwise when any data point that was z =  ± 3.29 from the paired sample mean difference score, resulting in the removal of two cases [35, 36].

An established benchmarking methodology was used to compare CAMC and PS outcomes with the RCT comparison. Effect sizes were calculated using the same formula for paired samples and standardised for comparison between the two samples, with the use of non-central t-tests and confidence intervals set to 95% [37, 38]. The non-central distribution was used to take account of the differences of power in the calculation of effect sizes according to sample size. It was assumed that standardised effect size values with non-overlapping confidence intervals were indicative of significant differences between the scaling and benchmark samples [39]. An effect size difference of d = 0.2 was considered to be clinically meaningful [40].

Analyses showed little systematic bias between participants providing data at both time points and those only completing Time 1 measures (see Appendix 2: Tables A1 and A2). Parents included in the analysis only differed by Time 1 CAMC scores and were more likely to be White British.

## Results

### Being a Parent reach, attendance and acceptability

The mean age for parents was 34.3 years, with 53.3% aged between 28–38 years and 20.3% aged between 21–27 years, see Table 1. The majority of parents were mothers (92.3%) and White British (67.9%). The largest minority group was South Asian (10.7%).

**Table 1:** Comparison of Scaling Programme and *Being a Parent* RCT parent demographic characteristics

Demographic characteristic	Value	Scaling Programme		RCT		Sig diff
		N	%	N	%	*p*
Parent gender	Female	648	92.3	56	96.6	n.s
Parent ethnicity	White British	452	67.9	13	22.4	< 0.05
English as a second language	Yes	159	23.9	27	46.6	< 0.05
Lone parent status	Yes	244	36.9	N/A	N/A	N/A
Parents highest qualification	University education completed	155	23.8	20	36.2	n.s
Type of housing	Owner/occupier	176	26.6	10	19.0	n.s
Work status	Unemployed	141	21.4	12	22.4	n.s

The majority of parents (*n* = 476, 75.3%) lived in areas with higher than UK average social deprivation. Over a quarter (27.4%, *n* = 173) lived in communities categorised in the 10% most socially deprived (33), 45.0% left school by age 16, 39.9% lived in rental accommodation, 21.4% were involuntarily unemployed and 36.9% were lone parents, see Table 1.

The only demographic differences between scaling and benchmark samples were ethnicity and first language, with a greater proportion of White British parents (Scaling sample = 67.9%; RCT sample = 22.4%, χ^2^(1) = 48.25, *p* < 0.05) and lower proportion of parents with English as a second language (Scaling sample = 23.9%; RCT sample = 46.6%, χ^2^(1) = 14.31, *p* < 0.05), see Table 1.

### Being a Parent attendance

Six hundred and eighty-four parents (73.5%) completed the BaP course across the 15 sites. This was significantly less than the 92.0% course completion rate achieved in the *Being a Parent* RCT (χ^2^(1) = 9.47, *p* < 0.01).

### Being a Parent acceptability

Parents reported high levels of satisfaction with course quality (TARS SQ), see Table 2. Scaling sample TARS SQ ratings were not significantly different from the benchmark comparison (Mean Scaling TARS SQ score = 18.8 (SD = 1.8), Mean RCT, TARS SQ score = 18.9, (SD = 1.4), t(453) = 0.4, *p* = ns).

**Table 2:** Scaling Programme TARS Course Satisfaction and Quality Results

Treatment Acceptability Rating Scale	Not at all	A little	Quite a lot	A great deal
Course Satisfaction & Quality				
Being a Parent group leader competence	0% (*n* = 0)	1.2% (*n* = 5)	20.5% (*n* = 83)	78.2% (*n* = 318)
Overall satisfaction with Being a Parent course	0.2% (*n* = 1)	0.7% (*n* = 3)	29.3% (*n* = 119)	69.7% (*n* = 283)
Being a Parent covered appropriate content/topics	0% (*n* = 0)	2.2% (*n* = 9)	24.2% (*n* = 98)	73.6% (*n* = 299)
Being a Parent group leaders communicated effectively	0% (*n* = 0)	0.5% (*n* = 2)	18.5% (*n* = 75)	81% (*n* = 329)
Being A Parent group leaders were motivating (e.g., energetic, attentive)	0% (*n* = 0)	1.5% (*n* = 6)	14.3% (*n* = 58)	84.2% (*n* = 342)
TARS Knowledge, Skills and Confidence				
Improved understanding of positive parenting	0% (*n* = 0)	5.2% (*n* = 21)	40.0% (*n* = 162)	54.8% (*n* = 223)
Increased use of positive parenting skills	0% (*n* = 0)	6.7% (*n* = 27)	38.7% (*n* = 157)	54.7% (*n* = 222)
Increased confidence in effective parenting	0.2% (*n* = 1)	9.4% (*n* = 38)	40.1% (*n* = 163)	50.2% (*n* = 204)
Commitment to use knowledge and skills gain from Being a Parent	0.2% (*n* = 1)	5.0% (*n* = 20)	39.5% (*n* = 160)	55.3% (*n* = 225)

Scaling sample parents reported high levels of knowledge, skills and confidence acquisition see Table 2. Despite these high levels of acquisition, TARS KSC mean score for the scaling sample was significantly lower than the benchmarking sample with an effect size suggesting a meaningful difference (Scaling TARS KSC Mean = 13.9 (SD = 2.1); RCT TARS KSC Mean = 14.6 (SD = 1.6), t(453) = 2.3, *p* < 0.05, d = 0.3).

### Parent reported concerns about child difficulties

Parent child concerns decreased significantly over time, equivalent to medium effect size (CAMC Total: Time 1 mean = 63.4, SD = 22.3, Time 2 mean = 48.6, SD = 25.3, t(338) = 9.3, *p* < 0.001, ES d = 0.6.), see Table 3, with reductions across all problem categories, except idiosyncratic parental concerns (CAMC Conduct Problem: Time 1 mean = 65.7, SD = 20.5, Time 2 mean = 50.8, SD = 25.5, t(197) = 7.5, *p* < 0.001, ES d = 0.6; CAMC Relationships: Time 1 mean = 59.9, SD = 15.1, Time 2 mean = 43.4, SD = 22.9, t(18) = 3.5, *p* < 0.01, ES d = 0.8. CAMC Child Self-Regulation: Time 1 mean = 68.2, SD = 21.2, Time 2 mean = 48.6, SD = 25.3, t(62) = 5.5, *p* < 0.001, ES d = 0.8. CAMC Child Emotional Distress: Time 1 mean = 55.8, SD = 26.3, Time 2 mean = 43.7, SD = 25.0, t(29) = 2.0, *p* < 0.05, ES d = 0.5. CAMC Other: Time 1 mean = 55.9, SD = 25.9, Time 2 mean = 45.2, SD = 23.3, t(19), *p* = n.s.).

**Table 3:** Scaling Programme child, parenting and parent well-being outcomes

Domain	N	Mean (SD) Time 1	Mean (SD) Time 2	Significance (*p*)	Effect size (d)
Parental mental well-being (SWEMWBS)	348	20.5 (3.5)	22.8 (3.8)	< 0.001	0.6
Parenting behaviour (PS)	348	3.5 (0.6)	3.0 (0.6)	< 0.001	0.9
Concerns about my child (CAMC)	339	63.4 (22.3)	48.6 (25.3)	< 0.001	0.6
Parenting Goals (PG)	310	36.9 (23.0)	69.1 (21.0)	< 0.001	1.2

Benchmarking comparison of confidence intervals between Scaling Programme CAMC Total effect size (d = 0.6, 95% CI = 0.48–0.76) and the equivalent RCT result (d = 0.85, 95% CI = 0.42–1.26) showed overlapping confidence intervals suggesting that the magnitude of CAMC improvement between the two samples was not significantly different. CAMC subscales were not used in the benchmark RCT.

### Parenting behaviour

Scaling programme parents reported significant improvements in positive parenting behaviour, equivalent to a large effect size, (PS Time 1 mean = 3.5, SD = 0.6, Time 2 mean = 3.0, SD = 0.6, t(347) = 14.1, *p* < 0.001, ES d = 0.9). Pre-course PS mean exceeded the established mean for clinic referred samples and below cut-off post-course. All three PS subscales significantly improved, ranging from small to medium effect sizes (PS Over-reactivity Time 1 mean = 3.0, SD = 0.9, Time 2 mean = 2.5, SD = 0.8, t(328) = 10.6, *p* < 0.001, ES d = 0.6; Verbosity Time 1 mean = 4.2, SD = 0.7, Time 2 mean = 3.9, SD = 0.7, t(350) = 8.4, *p* < 0.001, ES d = 0.4; Laxness Time 1 mean = 3.4, SD = 0.8, Time 2 mean = 3.0, SD = 0.7, t(330) = 10.0, *p* < 0.001 ES d = 0.6).

Scaling sample PS Total Score effect size (d = 0.9 (95% CI ADD CIs) and equivalent RCT result (RCT d = 0.8 (95% CI = 0.40–1.20) had overlapping confidence intervals, indicating no significant differences in the magnitude of improvement between the two samples.

### Parenting goals

Parents reported substantial progress towards achieving selected parenting goals, equivalent to a very large effect size (PG Time 1 mean = 36.9, SD = 23.0, Time 2 mean = 69.1, SD = 21.0, t(309) = -20.9, *p* < 0.001, ES d = 1.2). No RCT benchmarking information was available for comparison.

### Parental mental well-being

Parents starting Scaling Programme *Being a Parent* courses had a mean level of mental wellbeing below the UK national 25^th^ centile score of 21.5 [30]. Parents’ wellbeing significantly improved following the course equivalent to a medium effect size (Scaling SWEMWBS Time 1 Mean = 20.5, SD = 3.5SWEMWBS Time 2 mean = 22.8, SD = 3.8), t(347) = -11.0, *p* < 0.001, ES d = 0.6), with the Time 2 mean score similar to the UK national 50^th^ percentile. No RCT benchmarking information was available for comparison.

## Discussion

Large-scale real-world replication provides evidence about the extent to which efficacious interventions can maintain their performance outside of trial environments and without direct involvement of intervention developers. This study used a quasi-experimental design with embedded benchmarking to evaluate the scaling-up and scaling-out of the *Being a Parent* course in 15 newly established EPEC sites. Results provide indicative evidence to suggest that the new sites were probably able to replicate the demographic reach, parent acceptability and outcomes previously achieved under trial conditions.

Sites successfully engaged parents experiencing significant levels of social disadvantage, low levels of formal education and home ownership, together with significant levels of unemployment and lone parenthood. The scaling sample had a larger proportion of White British parents and fewer parents with English as a second language. This may have reflected ethnicity differences between the south London area in which the benchmark RCT took place and the population profiles of participating sites. For example, 19.5% of the UK population (16–65 years) is of Black and Minority Ethnic origin compared with 46.3% in the RCT area [41, 42]. Overall, the proportion of Black and Minority Ethnic parents participating in both EPEC trial and scaling programme was higher than local and national base rates.

The newly established EPEC sites’ mean course completion rates were substantially lower than the rate achieved under trial conditions. However, the mean parent course completion rate (73.5%) attained by sites was equivalent to mean completion rates reported by a wide range of profession-led behavioural parent training programmes [43]. Continued evaluation of completion rates over the long term will provide further understanding of the extent to which completion rates improve over time as sites become more experienced at parent recruitment, retention and intervention delivery or, for example, the extent to which completion rates vary according to site-specific conditions.

Participants reported high levels of acceptability and satisfaction with course content, methods and parent group leadership largely consistent with benchmarking data. The significant difference in TARS KSC subscale score potentially represents a modest reduction in some aspects of acceptability that might be attributed to the relative inexperience and unfamiliarity parent group leaders involved in the scaling programme, who were delivering Being a Parent courses for the first time. Longer term evaluation of TARS KSC will help to determine whether this relatively small difference persists. Parents also reported statistically significant and clinically meaningful improvements in parental wellbeing, parenting behaviour, parenting goal progress and child concerns. Where comparisons allowed, these improvements were largely similar to benchmarking data and consistent with those reported for comparable professionally-delivered interventions [12–14].

### Limitations

Conclusions about the replication at scale of this evidence-based approach should be drawn with a degree of caution. The study’s quasi-experimental design inevitably resulted in lower levels of internal validity compared with those typically achieved by randomised designs, particularly those using intention to treat analyses. Sample deterioration, missing data and analysis strategy may have had the effect of potentially inflating the magnitude of effect sizes reported.

The scaling programme consisted of multiple training and implementation components, many of which, though not all, were manualised and monitored for acceptability, fidelity and impact [31]. However, the current study was not designed to assess the relative impact of these multiple components nor site-related factors affecting performance [44]. It solely focussed on the reach, acceptability and impact of the manualised *Being a Parent* programme itself.

Benchmarking comparisons provided an efficient, pragmatic and low-cost method to compare scaling programme with trial performance. The study made direct benchmarking comparisons of most demographic and acceptability data though not all outcome measures. Ideally, benchmarking would have included the entire battery of RCT measures. This was not possible because of site concerns about undue real world measurement burden and site preference for the inclusion of the SWEMWBS, not used in the previous RCT. The benchmarking relied upon effect sizes derived from only one RCT. Though of high methodological quality, the benchmarking comparison would have been strengthened by access to a larger pool of trial results, narrowing effect size confidence intervals and reducing the likelihood of type 2 errors.

Data completion rates were at least comparable to similar scaling evaluations, possibly assisted by the emphasis on evaluation within each phase of the scaling implementation and the ongoing commitment of local site supervisors and parent group leaders. However, acceptability and impact data were not available for a significant minority of participants and results are therefore may be susceptible to bias [45].

Data acquisition methods typically used in trials, such as participants’ positive choice to engage in research, financial and altruistic incentivisation, and dedicated research staff were not available in this real-world evaluation because of cost constraints and delivery of the intervention as part of routine provision. The greater use of such methods within real world evaluation could potential improve the difficulties with data loss experienced in this and similar real world evaluations.

The exclusion of parents with insufficient English to complete measures means that it is unclear whether the impact and acceptability of the findings would be replicated with these populations. While one-third of the sample were from non-White British ethnicities and 23.9% of parents had English as a second language, conducting a post hoc analysis of differential outcome effects due to ethnicity would have been problematic given the sample’s ethnic heterogeneity.

The evaluation infrastructure built during the scaling programme means that EPEC is in a strong position to test data quality improvement methods including ongoing training, rapid and continuous site feedback, site-specific data improvement plan and cooperation on evaluation priorities across sites. Improvements in data quality and larger site samples will enable between-site acceptability and outcome comparisons, as well as the future examination of the effectiveness of specific scaling programme components.

## Conclusion

Despite methodological limitations, these findings suggest that it may be possible to successfully replicate at scale EPEC *Being a Parent*, a peer-led, community-based, parenting approach. Evaluation evidence suggests that reach, acceptability and impact were largely maintained across the cohort of 15 new sites. Successful replication of this peer-led approach at scale has considerable potential to make a significant contribution to improving child and parenting outcomes and, in so doing, reducing the child behaviour treatment gap, particularly for socially disadvantaged populations. The results also provide the basis to consider a large scale, definitive randomised trial of the scaling programme.

## Supplementary Information

**Additional file1: Figure A1.** EPEC Logic Model For Parent-Led, Group Format.

**Additional file 2.** Analysis for systematic bias of scaling programme participant non-responders.

## Data Availability

The datasets generated and/or analysed during the current study are not publicly available due privacy of participants involved but are available from the corresponding author on reasonable request.

## Abbreviations

NICE: National Institute for Health and Clinical Excellence
EPEC: Empowering Parents Empowering Communities
BaP: Being a Parent
NHS: National Health Service
EBI: Evidence based intervention
RCT: Randomised controlled trial
CAMC: Concern about my child measure
SWEMWBS: Short Warwick Edinburgh Mental Wellbeing Scale
PS: Parenting Scale
MPG: My Parenting Goals
TARS: Treatment Acceptability Rating Scale
ITT: Intention to treat
