# Supplementary material for: Impact of a peer-led, community-based parenting programme delivered at a national scale: an uncontrolled cohort design with benchmarking
Source: BMC Public Health. 2022 Jul 18;22:1377. doi: 10.1186/s12889-022-13691-y (PMC9295349; doi:10.1186/s12889-022-13691-y)
Supplement: Supplementary file 1 — Additional file1: Figure A1. EPEC Logic Model For Parent-Led, Group Format. [file 12889_2022_13691_MOESM1_ESM.docx]

# Appendix 1: Figure A1 EPEC Logic Model for parent-led, group format

Professional-led and delivered parenting programmes

Parent and family stigma, limited scope of services. Scale of need outweighs services ability to deliver.

Socially disadvantage and minority families are less likely to access, engage, attend and complete profession/service-led parenting interventions

Complex service pathways, signposting, wariness of EBIs, non- relational methods decrease access.

Poorer family outcomes, poor acceptability, inefficient and poor cost effectiveness

Inconsistent implementation of EBIs by professionals, lack of parent identification, reduced acceptability

Socially disadvantaged children are at increased risk of adverse

social and emotional developmental outcomes

Parenting, parent skills

EPEC Hub Functions

Effective manualised

Improved parent knowledge, skills and confidence,

and well being are key

mechanism to improve child outcomes and reduce risk

Evidence-based parenting

programmes are effective methods for improving parent and child outcomes

Effective community and parent

awareness, knowledge, access and engagement in parenting programmes

content, skilful

delivery, community locations, high attendance and acceptability

improved child S-E development, increased social capital, increased cost-effectiveness

Improved child,

parent and family outcomes

EPEC Hub team environment & shared leadership: belief in EPEC, emotional commitment to purpose/goals, collective learning/empowerment, social support, mutual participation and influence

**EPEC PARENT-LED PARENTING COURSES**

Improved social capital and community

Resilient parents &families are previous beneficiaries of EPEC

Effective identification,

selection, training and support of EPEC group leaders

EPEC Hub: Delivery in community locations by trained parent peers, effective ongoing engagement methods, effective group facilitation, fidelity and quality, supervision, outcome monitoring and reporting

outcomes


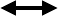


Socially disadvantaged children are at reduced risk of adverse social

and emotional developmental outcomes

Effective community and service systems to increase parent and community awareness, provide community locations, encourage parent and service engagement

Effective strategic and operation commissioning and support to promote peer-led components within local community and service networks
