# Supplementary material for: Impact of a peer-led, community-based parenting programme delivered at a national scale: an uncontrolled cohort design with benchmarking
Source: BMC Public Health. 2022 Jul 18;22:1377. doi: 10.1186/s12889-022-13691-y (PMC9295349; doi:10.1186/s12889-022-13691-y)
Supplement: Supplementary file 2 — Additional file 2. Analysis for systematic bias of scaling programme participant non-responders. [file 12889_2022_13691_MOESM2_ESM.docx]

**Appendix 2: Analysis for systematic bias of scaling programme participant non-responders**

Table A1: Time 1 outcome measure scores for parents included and not included in analysis

| Time 1 | Mean score parents included in analysis (n) | Mean score parents not included in analysis (n) | Difference |
| --- | --- | --- | --- |
| SWEMWBS | 20.5 (351) | 20.8 (287) | n.s |
| MPG | 36.9 (338) | 37.3 (265) | n.s |
| CAMC total score | 63.3 (342) | 57.8 (269) | p=0.03 |
| Total Parenting Scale | 3.5 (352) | 3.5 (272) | n.s |

*Note:* The sample size fluctuates per measure due to pairwise deletions of missing data.

Table A2: Time 1 demographic data for parents included and not included in the analysis

| Time 1 | Parents included in analysis (n) | Parents not included in analysis (n) | Difference |
| --- | --- | --- | --- |
| Parent age | 34.3 years (385) | 33.6 years (309) | n.s |
| Child age | 5.0 years (278) | 4.4 years (232) | n.s |
| Lone parent status | 37.5% (360) | 36.2% (301) | n.s |
| White British | 71.7% (361) | 63.3% (305) | p=0.02 |
| Left school before age 16 | 25.4% (354) | 25.6% (298) | n.s |
| Unemployed | 21.0% (357) | 21.9% (302) | n.s |

*Note:* The sample size fluctuates per measure due to pairwise deletions of missing data.
